# Genome Sequence of the Endophytic Bacterium *Bacillus thuringiensis* Strain KB1, a Potential Biocontrol Agent against Phytopathogens

**DOI:** 10.1128/genomeA.00279-16

**Published:** 2016-04-21

**Authors:** Haeyoung Jeong, Sung Hee Jo, Chi Eun Hong, Jeong Mee Park

**Affiliations:** a Super-Bacteria Research Center, Korea Research Institute of Bioscience and Biotechnology (KRIBB), Daejeon, Republic of Korea; b Biosystems and Bioengineering Program, University of Science and Technology (UST), Daejeon, Republic of Korea; c Molecular Biofarming Research Center, KRIBB, Daejeon, Republic of Korea

## Abstract

*Bacillus thuringiensis* is the most widely known microbial pesticide used in agricultural applications. Herein, we report a draft genome sequence of the endophytic bacterium *Bacillus thuringiensis* strain KB1, which exhibits antagonism against phytopathogens.

## GENOME ANNOUNCEMENT

*Bacillus thuringiensis* has been widely used as a biopesticide for decades owing to its production of insecticidal proteinaceous toxins. Endophytic colonization of *B. thuringiensis* in plants (1-4), even accompanied by the production of Cry proteins, is of special interest because it may be used for the development of pathogen-resistant crops (5). It had been shown that the endophytic strain KB1, originally isolated from the apoplastic fluid extracts of *Arabidopsis thaliana*, significantly increases the disease resistance of tomatoes to *Botrytis cinerea* and *Pseudomonas syringae* pv. tomato (6).

Genome sequencing was carried out using the Illumina HiSeq 2500 platform at the National Instrument Center for Environmental Management at Seoul National University (Seoul, Republic of Korea). A total of 861,644,958 bp of paired reads (151-cycle) were produced from a library with a fragment size of ca. 466 bp, which was constructed using a TruSeq Nano LT DNA sample preparation v2 kit. After adaptor sequence removal and quality trimming using Trimmomatic v0.32 (7), the reads were filtered to remove any possible contaminants or errors using khmer v2.0 (8). *De novo* genome assembly using CLC Genomics Workbench v8.5.1 generated 47 contigs with a 35.0% G+C content out of 441,060,070 bp of reads (with an average length of 136.9 bp). The total contig length, maximum contig length, and *N*_50_ were 5,748,443, 1,121,496, and 618,894 bp, respectively. Genome annotation using the Prokaryotic Genome Annotation Pipeline identified 5,666 coding sequences and 117 RNAs. Similar genomic regions with biosynthetic genes for bacillibactin and petrobactin were also identified using antiSMASH 3.0 (9).

KB1 was formerly classified into *B. cereus* based on a 16S rRNA gene sequence analysis (6). However, multilocus sequence analysis using the PubMLST resource (http://pubmlst.org/bcereus/) (10), which utilizes eight housekeeping genes for the typing of the *Bacillus cereus* group, revealed only one nucleotide difference at the *pyc* gene out of eight loci between KB1 and *B. thuringiensis* serovar *tochigiensis* HD868 (= BGSC 4Y1, GenBank CM000746.1) (11, 12). Moreover, specI analysis using 40 universal single-copy phylogenetic marker genes (13) and JSpecis analysis (14) showed that KB1 is most similar to HD868 (99.9% and 99.2% identity, respectively). Furthermore, the DNA-DNA hybridization estimate calculated using GGDC 2.2 (15) was 94.7%, which led us to tentatively reclassify KB1 as a *Bacillus thuringiensis* species. A putative plasmid sequence (contig_1, 154,083 bp) harboring a gene similar to *repS* of pXO2 was found without any other virulence-related genes. Crystal toxin gene was absent from the assembly, but a recent systemic study of the *B. cereus* group, which employed a whole-genome sequence-based approach in combination with classical gene-based typing methods (16), suggested that the presence or absence of pXO plasmids and *cry* genes cannot be used to discriminate species in the *B. cereus* group. Our genome data will help us understand the ecological role of KB1 and its potential use as a biocontrol agent, as well as its phylogenetic status.

### Nucleotide sequence accession numbers.

This whole-genome shotgun project has been deposited at DDBJ/ENA/GenBank under the accession number LSNJ00000000. The version described in this paper is version LSNJ01000000.
